# Modest Elevation in BNP in Asymptomatic Hypertensive Patients Reflects Sub-Clinical Cardiac Remodeling, Inflammation and Extracellular Matrix Changes

**DOI:** 10.1371/journal.pone.0049259

**Published:** 2012-11-12

**Authors:** Dermot Phelan, Chris Watson, Ramon Martos, Patrick Collier, Anil Patle, Seamas Donnelly, Mark Ledwidge, John Baugh, Ken McDonald

**Affiliations:** 1 Heart Failure Unit, St Vincent’s University Hospital, Elm Park, Dublin, Ireland; 2 School of Medicine and Medical Science, UCD Conway Institute, University College Dublin, Dublin, Ireland; Centro Cardiologico Monzino IRCCS, Italy

## Abstract

In asymptomatic subjects B-type natriuretic peptide (BNP) is associated with adverse cardiovascular outcomes even at levels well below contemporary thresholds used for the diagnosis of heart failure. The mechanisms behind these observations are unclear. We examined the hypothesis that in an asymptomatic hypertensive population BNP would be associated with sub-clinical evidence of cardiac remodeling, inflammation and extracellular matrix (ECM) alterations. We performed transthoracic echocardiography and sampled coronary sinus (CS) and peripheral serum from patients with low (n = 14) and high BNP (n = 27). Peripheral BNP was closely associated with CS levels (r = 0.92, p<0.001). CS BNP correlated significantly with CS levels of markers of collagen type I and III turnover including: PINP (r = 0.44, p = 0.008), CITP (r = 0.35, p = 0.03) and PIIINP (r = 0.35, p = 0.001), and with CS levels of inflammatory cytokines including: TNF-α (r = 0.49, p = 0.002), IL-6 (r = 0.35, p = 0.04), and IL-8 (r = 0.54, p<0.001). The high BNP group had greater CS expression of fibro-inflammatory biomarkers including: CITP (3.8±0.7 versus 5.1±1.9, p = 0.007), TNF-α (3.2±0.5 versus 3.7±1.1, p = 003), IL-6 (1.9±1.3 versus 3.4±2.7, p = 0.02) and hsCRP (1.2±1.1 versus 2.4±1.1, p = 0.04), and greater left ventricular mass index (97±20 versus 118±26 g/m^2^, p = 0.03) and left atrial volume index (18±2 versus 21±4, p = 0.008). Our data provide insight into the mechanisms behind the observed negative prognostic impact of modest elevations in BNP and suggest that in an asymptomatic hypertensive cohort a peripheral BNP measurement may be a useful marker of an early, sub-clinical pathological process characterized by cardiac remodeling, inflammation and ECM alterations.

## Introduction

B-type natriuretic peptide (BNP) is a cardioprotective hormone which is secreted by the left ventricle (LV) in response to wall stress and is involved in regulating volume homeostasis and cardiac remodeling [1], [2]. BNP is utilized in the clinical setting as a diagnostic tool to identify heart failure (HF) and LV dysfunction in addition to providing prognostic information in patients with HF, hypertension, and coronary artery disease [3], [4], [5], [6]. There is an emerging interest in its use in asymptomatic patients with cardiovascular risk factors [7], [8]. Wang *et. al.* demonstrated that BNP levels above the 80^th^ percentile (20 pg/ml for men and 23.3 pg/ml for women) in the Framingham Offspring Study was associated with a significant increased risk of major cardiovascular events including HF and death [7]. However, the underlying mechanism of the negative prognostic impact of a modest elevation in BNP in asymptomatic patients is unclear.

Arterial hypertension is an important preventable cause of premature death that is estimated to account for 4.5% of the total global disease burden [9]. Hypertensive heart disease (HHD) describes a process of remodeling which occurs in the myocardium when exposed to sustained elevation in arterial blood pressure. The two most notable features are myocyte hypertrophy and excessive extracellular matrix (ECM) collagen deposition, also termed “reactive fibrosis”. In physiological terms, increased fibrosis results in increased stiffness of the LV, diastolic dysfunction and if sufficiently severe, diastolic HF [10], [11]. Inflammation appears to be an important upstream stimulus for the development of reactive fibrosis [12], [13].

Several studies have shown a definite relationship between BNP and myocardial stiffness particularly in HHD [14], [15]. Furthermore, there is evidence from experimental and animal studies which show that natriuretic peptides are involved in regulating myocardial fibrosis [16], [17].

We hypothesized that a modest elevation in BNP levels in an asymptomatic hypertensive population would be associated with subclinical myocardial injury as determined by adverse cardiac remodeling and heightened expression of both inflammatory cytokines and serum markers of collagen metabolism. Coronary sinus serum was sampled as this localizes events to the myocardium and may be more sensitive to detect subtle changes in biomarker expression.

## Methods

### Participants and Protocol

We studied 41 Caucasian subjects with a history of hypertension who volunteered for the study. Patients were recruited prior to routine elective coronary catheterisation for suspected ischaemic chest pain. Patients had normal self-reported exercise capacity. At the time of recruitment patients were examined for signs of congestive cardiac failure/fluid overload which included assessment for: elevation in jugular venous pressure, basal pulmonary crepitations, a gallop rhythm (S3) or peripheral pitting oedema. Exclusion criteria included any findings suggestive of fluid overload, renal insufficiency (serum creatinine >1.5 mg/dl or estimated creatinine clearance (eCrCl) <45 ml/min as calculated using the Cockcroft-Gault formula), evidence of infection, conditions known to alter collagen turnover (including chronic liver disease, connective tissue disorders, metabolic bone diseases, and malignancy), conditions known to alter BNP levels (including HF, cardiomyopathy and recent ischemic events), and recent surgery or physical trauma (<6 months). were categorised into high and low BNP groups according to values above and below the 80^th^ percentile described in the Framingham Offspring Study (BNP 20 pg/ml for men and 23.3 pg/ml for women) [7]. All patients had appropriate clinical and laboratory evaluation to identify suitability for the study and exclusion criteria.

Office blood pressure monitoring was performed in the sitting position using a standard sphygmomanometer. Subjects underwent transthoracic echocardiography (TTE) and right heart catheterization for coronary sinus blood sampling within one week of each other. All subjects gave written informed consent to participate in the study. The Ethics Committee at St Vincent’s University Hospital approved the study protocol which conformed to the principles of the Helsinki Declaration.

### Coronary Sinus Blood Sampling

Following puncture of the right femoral vein, a 6F sheath was introduced using a Seldinger technique. A 5F catheter was positioned under fluoroscopic guidance to the os of the CS as confirmed by contrast injection. Peripheral blood samples were taken from the side arm of the femoral sheath and further samples from the CS os. Samples were collected into vacutainer tubes using ethylenediaminetetraacetic acid tubes for BNP and gel separation and clot activator tubes for biomarker determination. Samples were immediately stored at 4°C. Within 1 hour the serum tubes were centrifuged at 1500 rpm for 5 minutes at 4°C to separate off the serum. Serum was then aliquoted out into small volume microcentrifuge tubes and stored in a −80°C freezer until use.

### Echocardiography

TTE was performed using a Philips IE33 by a single experienced echocardiographer. Measurements were performed by a single reader blinded to BNP results. The following measurements were performed as per ASE guidelines [18]: ejection fraction (EF) using the biplane Simpson’s method from apical 2- and 4- chamber windows, left atrial volume index (LAVI) using a biplane area-length formula, end-diastolic inter-ventricular septal (IVS) and posterior wall (PW) thickness and LV internal dimension in diastole (LVIDd). LV mass index (LVMI) was calculated based on modeling the LV as a prolate ellipse [18]. Diastolic parameters including peak early (E) and late (A) diastolic mitral inflow velocity and its ratio (E/A), deceleration time (DT) and average of the medial and lateral mitral annular diastolic velocities (e’) were also measured according to ASE guidelines [19]. All echocardiography data were based on the mean of 3 measurements on consecutive cardiac cycles.

### Serum Biomarker Assessment

BNP was quantified using a Triage meter BNP assay (assay sensitivity <5 ng/ml) (Biosite Inc.).Serum procollagen type I amino-terminal (assay sensitivity 13.0 ng/ml) (PINP), carboxy-terminal (assay sensitivity 2.0 ng/ml) (PICP), carboxy telopeptide of type I collagen (assay sensitivity 0.5 ng/ml) (CITP) and procollagen type III amino-terminal (assay sensitivity 1.9 ng/ml) (PIIINP) were quantified using radioimmunoassay (Orion Diagnostica). A custom made ultrasensitive Meso Scale Discovery (MSD) 4-Plex ELISA enabled simultaneous detection of interleukin (IL)-6, IL-8 and tumour necrosis factor alpha (TNFα) using an MSD Sector Imager 2400 reader. For each of these inﬂammatory markers, the assay sensitivity of was ≤0.7 pg/ml. Duplicate measurements were performed in all the above tests. High sensitivity CRP (hsCRP) serum concentrations were also determined using ELISA (MSD). All assays were carried out according to manufacturer’s guidelines or as described previously [20].

### Statistical Analysis

For continuous variables, summary statistics are presented as the mean ± standard deviation (SD) or median and 25–75^th^ percentiles. Categorical variables are presented as frequencies and percentages (in parenthesis). Comparisons between the low and high BNP groups were made using independent t-test, Welsh test, or chi square test where appropriate. The relationships between BNP, markers of collagen turnover and markers of inflammation were assessed using Pearson’s correlation coefficient for variables that were approximately normally distributed. In addition, Pearson’s correlation coefficient was used with log-transformation of variables with non-normal distribution. A p value of <0.05 was considered statistically significant.

## Results

### Participant Characteristics

Baseline characteristics of the participants are presented in table 1. The mean age of participants was 65±9 years and 44% were male. While 71% were taking either an angiotensin converting enzyme inhibitor (ACEi) or angiotensin receptor blocker (ARB) and 54% were taking a β-blocker, blood pressure (BP) control remained sub-optimal (mean±SD systolic BP/diastolic BP, 150±19/77±10 mmHg). No patient was taking both an ACEi and an ARB. 14 and 27 patients were categorised into low and high BNP groups respectively. The only significant difference between the two groups was in the usage of β-blockers (29% versus 66%, p = 0.03, low versus high BNP groups respectively). Of note, there was no significant difference between the two groups in terms of the number of patients taking both an ACEi/ARB and a β-blocker (29% versus 52%, p = 0.27, low versus high BNP groups respectively), or in terms of the number of patients taking two or more medications (43% versus 67%, p = 0.15, low versus high BNP groups respectively).

**Table 1:** Baseline characteristics of the study participants.

	Total Population (n = 41)	Low BNP (n = 14)	High BNP (n = 27)	*P*
Age, yr	65±9	63.2±9.4	66.6±9.4	0.28
Gender, male	18 (44)	7 (50)	11 (41)	0.81
BMI, kg/m^2^	28±6	27.5±7.2	28.2±5.7	0.74
SBP, mmHg	150±19	146±18	151±19	0.38
DBP, mmHg	77±10	75±11	78±11	0.4
PP, mmHg	72±18	71±18	73±19	0.69
Diabetes	10 (24)	3 (21)	7 (26)	0.99
**Biochemical parameters**				
Sodium, mmol/l	138±4	138±4	139±3	0.35
Potassium, mmol/l	4.2±0.4	4.1±0.3	4.3±0.4	0.14
Urea, mmol/l	6.2±1.7	6.1±1.1	6.3±2.0	0.62
Creatinine, mg/dl	1.03±0.27	1.04±0.24	1.02±0.28	0.78
Hemoglobin, g/dl	13.1±1.9	12.6±2.1	13.4±1.7	0.21
eCrCl, ml/min	78±29	81±36	77±26	0.7
Calcium, mg/dl	9.1±0.5	9.0±0.6	9.2±0.5	0.42
Alkaline Phosphatase, U/L	77±30	80±3.2	76±28	0.72
**Medications**				
ACEi/ARB	29 (71)	9 (64)	20 (74)	0.72
β-blocker	22 (54)	4 (29)	18 (66)	0.03
Statin	25 (61)	9 (64)	16 (59)	0.99

Data are presented as mean ± SD or as number (percentage).

BMI = body mass index; SBP = systolic blood pressure; DBP = diastolic blood pressure; PP = pulse pressure; eCrCl = estimated creatinine clearance; ACEi = angiotensin converting enzyme inhibitor; ARB = angiotensin receptor antagonist.

### Echocardiographic Characteristics

Table 2 shows the echocardiographic parameters of the study participants. Both LVMI (97±20 versus 118±26 g/m^2^, p = 0.03) and LAVI (18±2 versus 21±4, p = 0.008) were significantly higher in the high BNP group when compared to the low BNP group. There were no other significant differences between the two groups.

**Table 2:** Echocardiographic measurements.

	Total Population (n = 41)	Low BNP n = 14	High BNP n = 27	*P*
Ejection Fraction, %	65±9	67±11	64±9	0.41
IVS, mm	11.5±2.6	11.1±1.7	11.7±2.9	0.62
PW, mm	9.3±2	9.0±1.1	9.5±2.3	0.52
LVIDd, mm	52±5	51±6	53±4	0.29
LVMI, g/m^2^	114±28	97±20	118±26	0.03
Peak E, cm/s	75±20	70±17	78±22	0.23
Peak A, cm/s	81±25	76±23	83±26	0.42
EA	0.96±0.3	0.97±0.3	0.95±0.3	0.91
DT, ms	238±55	238±45	238±59	0.99
e’, cm/s	11±3	11±4	10±2	0.33
E/e’	7.2±2.3	6.7±2.4	7.4±2.2	0.34
LAVI, ml/m^2^	20±4	18±2	21±4	0.008

Data are presented as mean ± SD.

LVMI = left ventricular mass index; IVS = intraventricular septum; PW = posterior wall; LVIDd = left ventricular internal dimension in diastole; DT = E-wave deceleration time; IVRT = isovolumic relaxation time; LAVI = LA volume index.

### Serum Biomarker Expression between Groups

In samples collected from the periphery, only hsCRP was significantly different between the two groups (1.1±1.4 versus 2.7±2.7, p = 0.4, low versus high BNP groups respectively) (table 3). CS samples revealed greater expression of a number of fibro-inflammatory biomarkers in the high BNP group including: CITP (3.8±0.7 versus 5.1±1.9, p = 0.007), TNF-α (3.2±0.5 versus 3.7±1.1, p = 003), IL-6 (1.9±1.3 versus 3.4±2.7, p = 0.02) and hsCRP (1.2±1.1 versus 2.4±1.1, p = 0.04).

**Table 3:** Comparison of biomarker levels from peripheral and coronary sinus samples in low and high BNP groups.

	Peripheral Serum Samples (n = 41)			Coronary Sinus Serum Samples (n = 41)		
	Low BNP (n = 14)n = 14	High BNP (n = 27)n = 27	*P*	Low BNP (n = 14)n = 14	High BNP (n = 27)n = 27	*P*
BNP,pg/mL	10.4 (9.4,14.1)	58 (32,129)	<0.001	24 (9.8,42.3)	115 (59,403)	<0.001
PICP,ng/mL	272±43	267±4	0.74	331±107	297±10	0.39
PINP,mg/mL	27.5±14.3	35.3±12.9	0.266	27.5±7.8	32.4±12.8	0.179
CITP,µg/L	3.3±1.1	4.9±3.7	0.06	3.8±0.7	5.1±1.9	0.007
PIIINP,µg/mL	2.7±1.4	2.2±0.8	0.30	2.4±0.5	2.8±1.0	0.11
TNFα,pg/mL	4.0±2.0	3.7±1.1	0.63	3.2±0.5	3.7±1.1	0.03
IL-6,pg/mL	1.2±1.0	1.9±1.6	0.13	1.9±1.3	3.4±2.7	0.02
IL-8, pg/mL	6.7±2.7	6.5±3.2	0.86	5.7±1.7	6.4±3.6	0.46
hsCRP,mg/L	1.4±1.1	2.7±2.7	0.04	1.2±1.1	2.4±1.1	0.04

Data are presented mean ± standard deviation or as median and 25th and 75th percentiles in parenthesis. BNP = B-type natriuretic peptide; IL = interleukin; hsCRP high sensitivity C-reactive protein; TNFα = tumor necrosis factor-α; PICP = carboxy-terminal; PINP = amino-terminal; CITP = carboxy-terminal telopeptide of procollagen type I; PIIINP = amino-terminal propeptide of procollagen type III.

#### BNP, fibro-inflammatory biomarkers and cardiac remodeling

Peripheral BNP was very closely associated with CS levels (r = 0.92, p<0.001).

**Figure 1:**
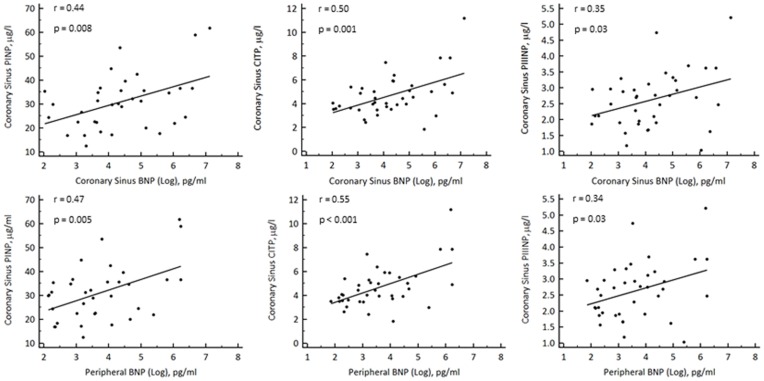
Scatter plots showing significant correlations between levels of BNP (log transformed) in serum from both the coronary sinus and periphery and levels of markers of collagen type I and type III turnover including PINP, CITP and PIIINP in serum from the coronary sinus.

CS BNP correlated significantly with CS levels of markers of collagen type I and III turnover including: PINP (r = 0.44, p = 0.008), CITP (r = 0.35, p = 0.03) and PIIINP (r = 0.35, p = 0.001). Furthermore, peripheral BNP also correlated significantly with CS PINP (r = 0.47, p = 0.005), CITP (r = 0.55, p<0.001) and PIIINP (r = 0.34, p = 0.03) (Figure 1).

**Figure 2:**
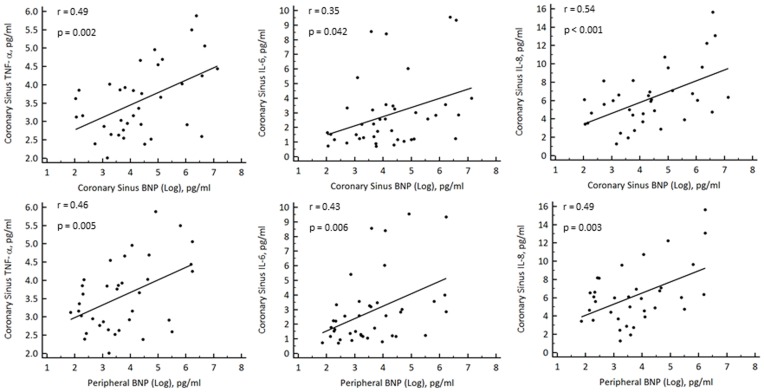
Scatter plots showing significant correlations between levels of BNP (log transformed) in serum from both the coronary sinus and periphery and the inflammatory cytokines TNF-α, IL-6 and IL-8 in serum from the coronary sinus.

Both CS and peripheral BNP correlated significantly with CS levels of inflammatory cytokines including: TNF-α (r = 0.49, p = 0.002 and r = 0.43, p = 0.006 for CS and peripheral BNP respectively), IL-6 (r = 0.35, p = 0.04 and r = 0.49, p = 0003), and IL-8 (r = 0.54, p<0.001 and r = 48, p = 0.003) (Figure 2).

**Figure 3:**
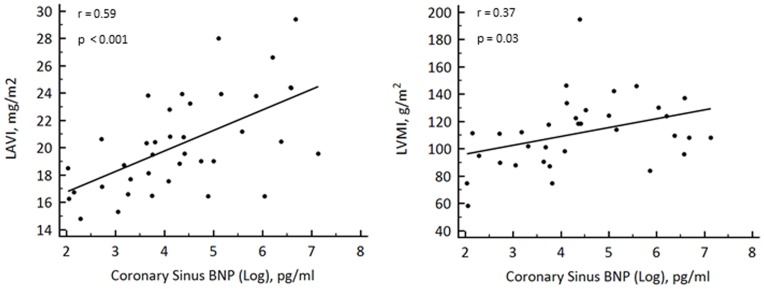
Scatter plots showing significant correlations between CS levels of BNP (log transformed) and structural changes in the heart including LAVI and LVMI.

In this population CS BNP was significantly associated with LAVI (r = 0.59, p<0.001) and LVMI (r = 0.37, p = 0.003)(Figure 3).

## Discussion

The present cross-sectional study demonstrated that in an asymptomatic hypertensive population a modest elevation in BNP was associated with increased expression of fibro-inflammatory biomarkers in serum sampled from the coronary sinus. A modest elevation in BNP is associated with increased LVMI and LAVI. Furthermore, significant correlations exist between markers of inflammation, collagen turnover and BNP.

### Hypertension, Reactive Fibrosis and BNP

It is estimated that between 25 and 30% of the adult population in the USA have hypertension [21]. It plays a major etiological role in the development of HF and ischaemic heart disease, with even modest increases in blood pressure contributing to the long term risk [9], [22]. The relationship between reactive fibrosis and hypertension is well described. Studies examining the collagen volume fraction of post-mortem hearts and endomyocardial biopsies demonstrate excessive accumulation of ECM collagen in patients with a history of hypertension and left ventricular hypertrophy (LVH) [23], [24], [25]. Indeed, evidence of excessive interstitial fibrosis has been demonstrated even in the early phases of hypertension in patients with only mild LVH [24], [26]. These observations have been directly linked to abnormalities in diastolic and systolic function [25], [27]. Furthermore, regression of the degree of fibrosis results in improvement in LV function [28], [29]. Therefore, the diagnosis of early changes in myocardial collagen content is of clinical relevance. However, taking endomyocardial biopsies in asymptomatic patients is unjustifiable and imaging techniques may be inadequate due to lack of sensitivity, technical and interpretive difficulties, dependence on loading conditions, and limitations in the presence of conditions such as atrial fibrillation [30], [31]. To address this issue, circulating terminal peptides which are cleaved and released into the serum during collagen type I synthesis (PICP, PINP)and degradation (CITP ) and collagen type III synthesis and degradation (PIIINP) have been examined and validated as markers of myocardial collagen content and function [20], [27], [32], [33]. However, there are a number of difficulties with the practical utility of these biomarkers. First, they are not specific to the myocardium. Second, their validation excluded subjects with rheumatological, hepatic or malignant disease and finally they are not routinely measured in clinical practice. In contrast, BNP is cardiac specific, extensively validated in unrestricted populations and widely available. The correlations observed in our study suggest that peripheral BNP may be a useful surrogate of markers of collagen turnover as detected in the coronary sinus. This is consistent with data showing BNP’s association with cardiovascular stiffening [14], [15].

In the high BNP group we observed a significant increase in CITP expression when compared to the low BNP group suggesting a predominance of collagen type I catabolism. These data may appear counterintuitive in the context of increased LVMI observed in this group. However, previous studies have demonstrated an increase in CITP in HHD and have suggested that this may be an early indication of degradation in the ECM scaffolding with resultant progression to chamber dilatation [27], [33], [34]. Despite LAVI measuring within normal limits in both groups, we noted a significant increase in the high BNP group (18±2 versus 21±4 ml/m^2^, p = 0.008). It is possible that the observed elevation in CITP is as a result of this early LA remodeling.

It is interesting to note that the BNP cut-off identified previously and used in this study divides our population into LAVI groups similar to those used by Ristow *et. al.,* where an increased risk of HF in subjects with LAVI measurements of 20–30 ml/m^2^ versus <20 ml/m^2^ was demonstrated. Consistent with the negative prognostic attributes of both BNP and LAVI, CITP has also been associated with adverse cardiac events [35].

### Hypertension, Inflammation and BNP

Increasing evidence suggests that inflammation may play a key role in the pathophysiology of hypertension [36]. A common event in many models of hypertension and cardiac fibrosis is heightened myocardial expression of pro-inflammatory cytokines [37], [38]. Melendez *et. al.* recently demonstrated a significant increase in myocardial collagen volume fraction, ventricular stiffness and concentric LVH in rats infused with IL-6 [37]. TNF-α has been directly implicated in the development of myocardial fibrosis [38], [39]. IL-8 is a potent neutrophil chemokine that is involved in the early inflammatory responses prior to replacement fibrosis [40]. Finally CRP, the most investigated cytokine in hypertension, has been repeatedly implicated in both the initiation and progression of the disorder [36]. Previous work has demonstrated an association between BNP and inflammatory cytokines. Mechanistically, BNP has immunomodulatory activity on macrophages and directly regulates the production of major inflammatory cytokines including TNF- α [41]. A positive feedback mechanism may exist, as TNF-α has been shown to induce secretion of BNP from cardiomyocytes *in vitro* [42], [43]. While these data are relatively new, taken together, they suggest an interdependent relationship between BNP and inflammatory cytokines in the progression of cardiac interstitial fibrosis. In our data, the correlations observed between BNP and measured cytokines support this concept and suggest that peripheral BNP may be a useful indicator of myocardial injury.

### Clinical Implications and limitations

The precise clinical implications of these observations are uncertain and it is not yet clear how best to manage asymptomatic hypertensive patients with a modestly elevated BNP. The prognostic evidence of elevated cardiovascular risk associated with such patients at a minimum underline the need for close attention to risk factor management. The observation that elevated BNP in hypertensive patients is associated with abnormal nocturnal blood pressure patterns underlines the need to completely assess blood pressure control [44]. There may also be a strong argument for the use of therapies which have been shown to regulate markers of myocardial interstitial disease such as agents that modulate the renin angiotensin aldosterone system [28], [29]. Furthermore, the Jupiter trial has shown a potential impact of anti-inflammatory effects of statin therapy in improving outcome in a similar patient population as the high BNP group [45]. It is possible that some of these benefits were mediated by the impact of rosuvastatin on myocardial fibrosis, a downstream result of inflammation.

In interpreting these data certain limitations need to be considered. First, we have used serum markers of collagen turnover taken from the CS as a surrogate of myocardial interstitial disease without endomyocardial biopsy evidence of fibrosis. Second, although BNP is a robust predictor of cardiovascular outcome in an unselected patient population [7], [8]_ENREF_4 our population excluded patients with diseases that alter collagen and inflammatory markers. Third, we note the increased usage of beta-blockers in the elevated BNP cohort. There is some evidence that beta-blocker therapy can modulate BNP levels, although variable effects are seen dependent upon the patient cohort and the duration of therapy [46], [47]. In addition, as there is clinical and experimental evidence that some beta-blocker therapies have anti-inflammatory [48], [49] and anti-fibrotic [49], [50] effects we postulate that the levels of markers of inflammation and fibrosis may have been even higher in elevated BNP group had they not been taking beta-blockers. However, the true relationship between beta-blocker therapy, BNP expression, inflammation and fibrosis warrants further detailed study. Finally, blood pressure readings were taken from a single reading and by multiple operators which may have resulted in an inaccurate representation of the overall blood pressure control. For this reason no correlations were performed between blood pressure reading s and any of the biomarkers measured.

### Conclusions

Risk stratification allows the physician to identify those patients who will benefit most from more aggressive surveillance and management. For asymptomatic hypertensive patients a means of risk stratification which is rapid, readily available, relatively inexpensive and capable of providing information beyond a history and physical examination is of particular importance. In this regard, BNP is a useful marker of adverse outcomes in asymptomatic patients even at levels well below the contemporary thresholds used in the diagnosis of HF. Our data provide insight into the mechanisms behind these observations and suggest that in an asymptomatic hypertensive cohort a peripheral BNP measurement may be a useful marker of an early, sub-clinical pathological process characterized by cardiac remodeling, inflammation and extracellular matrix turnover.

## References

[1] Nishikimi T, Maeda N, Matsuoka H (2006) The role of natriuretic peptides in cardioprotection. Cardiovasc Res 69: 318–328. 16289003 10.1016/j.cardiores.2005.10.001

[2] Levin ER, Gardner DG, Samson WK (1998) Natriuretic peptides. N Engl J Med 339: 321–328. 9682046 10.1056/NEJM199807303390507

[3] Maisel AS, Krishnaswamy P, Nowak RM, McCord J, Hollander JE, et al (2002) Rapid measurement of B-type natriuretic peptide in the emergency diagnosis of heart failure. N Engl J Med 347: 161–167. 12124404 10.1056/NEJMoa020233

[4] Cheng V, Kazanagra R, Garcia A, Lenert L, Krishnaswamy P, et al (2001) A rapid bedside test for B-type peptide predicts treatment outcomes in patients admitted for decompensated heart failure: a pilot study. J Am Coll Cardiol 37: 386–391. 11216951 10.1016/s0735-1097(00)01157-8

[5] Omland T, Sabatine MS, Jablonski KA, Rice MM, Hsia J, et al (2007) Prognostic value of B-Type natriuretic peptides in patients with stable coronary artery disease: the PEACE Trial. J Am Coll Cardiol 50: 205–214. 17631211 10.1016/j.jacc.2007.03.038

[6] Paget V, Legedz L, Gaudebout N, Girerd N, Bricca G, et al (2011) N-terminal pro-brain natriuretic peptide: a powerful predictor of mortality in hypertension. Hypertension 57: 702–709. 21383312 10.1161/HYPERTENSIONAHA.110.163550

[7] Wang TJ, Larson MG, Levy D, Benjamin EJ, Leip EP, et al (2004) Plasma natriuretic peptide levels and the risk of cardiovascular events and death. N Engl J Med 350: 655–663. 14960742 10.1056/NEJMoa031994

[8] Tsuchida K, Tanabe K (2008) Plasma brain natriuretic peptide concentrations and the risk of cardiovascular events and death in general practice. J Cardiol 52: 212–223. 19027599 10.1016/j.jjcc.2008.07.007

[9] Whitworth JA (2003) 2003 World Health Organization (WHO)/International Society of Hypertension (ISH) statement on management of hypertension. J Hypertens 21: 1983–1992. 14597836 10.1097/00004872-200311000-00002

[10] Diez J, Laviades C, Mayor G, Gil MJ, Monreal I (1995) Increased serum concentrations of procollagen peptides in essential hypertension. Relation to cardiac alterations. Circulation 91: 1450–1456. 7867186 10.1161/01.cir.91.5.1450

[11] Lopez B, Gonzalez A, Varo N, Laviades C, Querejeta R, et al (2001) Biochemical assessment of myocardial fibrosis in hypertensive heart disease. Hypertension 38: 1222–1226. 11711527 10.1161/hy1101.098549

[12] Kuwahara F, Kai H, Tokuda K, Takeya M, Takeshita A, et al (2004) Hypertensive myocardial fibrosis and diastolic dysfunction: another model of inflammation? Hypertension 43: 739–745. 14967845 10.1161/01.HYP.0000118584.33350.7d

[13] Kai H, Kuwahara F, Tokuda K, Imaizumi T (2005) Diastolic dysfunction in hypertensive hearts: roles of perivascular inflammation and reactive myocardial fibrosis. Hypertens Res 28: 483–490. 16231753 10.1291/hypres.28.483

[14] Watanabe S, Shite J, Takaoka H, Shinke T, Imuro Y, et al (2006) Myocardial stiffness is an important determinant of the plasma brain natriuretic peptide concentration in patients with both diastolic and systolic heart failure. Eur Heart J 27: 832–838. 16464912 10.1093/eurheartj/ehi772

[15] Chatzis D, Tsioufis C, Tsiachris D, Taxiarchou E, Lalos S, et al. (2009) Brain natriuretic peptide as an integrator of cardiovascular stiffening in hypertension. Int J Cardiol. 10.1016/j.ijcard.2008.12.018 19157602

[16] Kapoun AM, Liang F, O’Young G, Damm DL, Quon D, et al (2004) B-type natriuretic peptide exerts broad functional opposition to transforming growth factor-beta in primary human cardiac fibroblasts: fibrosis, myofibroblast conversion, proliferation, and inflammation. Circ Res 94: 453–461. 14726474 10.1161/01.RES.0000117070.86556.9F

[17] Tamura N, Ogawa Y, Chusho H, Nakamura K, Nakao K, et al (2000) Cardiac fibrosis in mice lacking brain natriuretic peptide. Proc Natl Acad Sci U S A 97: 4239–4244. 10737768 10.1073/pnas.070371497 PMC18212

[18] Lang RM, Bierig M, Devereux RB, Flachskampf FA, Foster E, et al (2005) Recommendations for chamber quantification: a report from the American Society of Echocardiography’s Guidelines and Standards Committee and the Chamber Quantification Writing Group, developed in conjunction with the European Association of Echocardiography, a branch of the European Society of Cardiology. J Am Soc Echocardiogr 18: 1440–1463. 16376782 10.1016/j.echo.2005.10.005

[19] Nagueh SF, Appleton CP, Gillebert TC, Marino PN, Oh JK, et al (2009) Recommendations for the evaluation of left ventricular diastolic function by echocardiography. J Am Soc Echocardiogr 22: 107–133. 19187853 10.1016/j.echo.2008.11.023

[20] Martos R, Baugh J, Ledwidge M, O’Loughlin C, Conlon C, et al (2007) Diastolic heart failure: evidence of increased myocardial collagen turnover linked to diastolic dysfunction. Circulation 115: 888–895. 17283265 10.1161/CIRCULATIONAHA.106.638569

[21] Hajjar I, Kotchen TA (2003) Trends in prevalence, awareness, treatment, and control of hypertension in the United States, 1988–2000. Jama 290: 199–206. 12851274 10.1001/jama.290.2.199

[22] Levy D, Larson MG, Vasan RS, Kannel WB, Ho KK (1996) The progression from hypertension to congestive heart failure. Jama 275: 1557–1562. 8622246

[23] Tanaka M, Fujiwara H, Onodera T, Wu DJ, Hamashima Y, et al (1986) Quantitative analysis of myocardial fibrosis in normals, hypertensive hearts, and hypertrophic cardiomyopathy. Br Heart J 55: 575–581. 3718796 10.1136/hrt.55.6.575 PMC1236764

[24] Rossi MA (1998) Pathologic fibrosis and connective tissue matrix in left ventricular hypertrophy due to chronic arterial hypertension in humans. J Hypertens 16: 1031–1041. 9794745 10.1097/00004872-199816070-00018

[25] Querejeta R, Lopez B, Gonzalez A, Sanchez E, Larman M, et al (2004) Increased collagen type I synthesis in patients with heart failure of hypertensive origin: relation to myocardial fibrosis. Circulation 110: 1263–1268. 15313958 10.1161/01.CIR.0000140973.60992.9A

[26] Ciulla M, Paliotti R, Hess DB, Tjahja E, Campbell SE, et al (1997) Echocardiographic patterns of myocardial fibrosis in hypertensive patients: endomyocardial biopsy versus ultrasonic tissue characterization. J Am Soc Echocardiogr 10: 657–664. 9282355 10.1016/s0894-7317(97)70028-2

[27] Plaksej R, Kosmala W, Frantz S, Herrmann S, Niemann M, et al (2009) Relation of circulating markers of fibrosis and progression of left and right ventricular dysfunction in hypertensive patients with heart failure. J Hypertens 27: 2483–2491. 19887955 10.1097/HJH.0b013e3283316c4d

[28] Diez J, Querejeta R, Lopez B, Gonzalez A, Larman M, et al (2002) Losartan-dependent regression of myocardial fibrosis is associated with reduction of left ventricular chamber stiffness in hypertensive patients. Circulation 105: 2512–2517. 12034658 10.1161/01.cir.0000017264.66561.3d

[29] Brilla CG, Funck RC, Rupp H (2000) Lisinopril-mediated regression of myocardial fibrosis in patients with hypertensive heart disease. Circulation 102: 1388–1393. 10993857 10.1161/01.cir.102.12.1388

[30] Grodecki PV, Klein AL (1993) Pitfalls in the echo-Doppler assessment of diastolic dysfunction. Echocardiography 10: 213–234. 10148406 10.1111/j.1540-8175.1993.tb00032.x

[31] Oguzhan A, Arinc H, Abaci A, Topsakal R, Kemal Eryol N, et al (2005) Preload dependence of Doppler tissue imaging derived indexes of left ventricular diastolic function. Echocardiography 22: 320–325. 15839987 10.1111/j.1540-8175.2005.03177.x

[32] Querejeta R, Varo N, Lopez B, Larman M, Artinano E, et al (2000) Serum carboxy-terminal propeptide of procollagen type I is a marker of myocardial fibrosis in hypertensive heart disease. Circulation 101: 1729–1735. 10758057 10.1161/01.cir.101.14.1729

[33] Collier P, Watson CJ, Voon V, Phelan D, Jan A, et al. (2011) Can emerging biomarkers of myocardial remodelling identify asymptomatic hypertensive patients at risk for diastolic dysfunction and diastolic heart failure? Eur J Heart Fail. 10.1093/eurjhf/hfr079 21719449

[34] Berk BC, Fujiwara K, Lehoux S (2007) ECM remodeling in hypertensive heart disease. J Clin Invest 117: 568–575. 17332884 10.1172/JCI31044 PMC1804378

[35] Barasch E, Gottdiener JS, Aurigemma G, Kitzman DW, Han J, et al (2011) The relationship between serum markers of collagen turnover and cardiovascular outcome in the elderly: the Cardiovascular Health Study. Circ Heart Fail 4: 733–739. 21900186 10.1161/CIRCHEARTFAILURE.111.962027 PMC3263368

[36] Montecucco F, Pende A, Quercioli A, Mach F (2011) et.al (2011) Inflammation in the pathophysiology of essential hypertension. J Nephrol. 24(1): 23–34. 10.5301/jn.2010.4729 20437401

[37] Melendez GC, McLarty JL, Levick SP, Du Y, Janicki JS, et al (2010) Interleukin 6 mediates myocardial fibrosis, concentric hypertrophy, and diastolic dysfunction in rats. Hypertension 56: 225–231. 20606113 10.1161/HYPERTENSIONAHA.109.148635 PMC2921860

[38] Sun M, Chen M, Dawood F, Zurawska U, Li JY, et al (2007) Tumor necrosis factor-alpha mediates cardiac remodeling and ventricular dysfunction after pressure overload state. Circulation 115: 1398–1407. 17353445 10.1161/CIRCULATIONAHA.106.643585

[39] Sivasubramanian N, Coker ML, Kurrelmeyer KM, MacLellan WR, DeMayo FJ, et al (2001) Left ventricular remodeling in transgenic mice with cardiac restricted overexpression of tumor necrosis factor. Circulation 104: 826–831. 11502710 10.1161/hc3401.093154

[40] Frangogiannis NG (2004) Chemokines in the ischemic myocardium: from inflammation to fibrosis. Inflamm Res 53: 585–595. 15693606 10.1007/s00011-004-1298-5

[41] Chiurchiu V, Izzi V, D’Aquilio F, Carotenuto F, Di Nardo P, et al (2008) Brain Natriuretic Peptide (BNP) regulates the production of inflammatory mediators in human THP-1 macrophages. Regul Pept 148: 26–32. 18410972 10.1016/j.regpep.2008.02.009

[42] de Bold AJ (2009) Cardiac natriuretic peptides gene expression and secretion in inflammation. J Investig Med 57: 29–32. 10.2310/JIM.0b013e3181948b37 19158604

[43] Shor R, Rozenman Y, Bolshinsky A, Harpaz D, Tilis Y, et al (2006) BNP in septic patients without systolic myocardial dysfunction. Eur J Intern Med 17: 536–540. 17142170 10.1016/j.ejim.2006.07.013

[44] Nakatsu T, Shinohata R, Mashima K, Yuki Y, Nishitani A, et al (2007) Use of plasma B-type natriuretic peptide level to identify asymptomatic hypertensive patients with abnormal diurnal blood pressure variation profiles: nondippers, extreme dippers, and risers. Hypertens Res 30: 651–658. 17785934 10.1291/hypres.30.651

[45] Ridker PM, Danielson E, Fonseca FA, Genest J, Gotto AM Jr, et al (2008) Rosuvastatin to prevent vascular events in men and women with elevated C-reactive protein. N Engl J Med 359: 2195–2207. 18997196 10.1056/NEJMoa0807646

[46] Troughton RW, Richards AM, Yandle TG, Frampton CM, Nicholls MG (2007) The effects of medications on circulating levels of cardiac natriuretic peptides. Ann Med 39: 242–260. 17558597 10.1080/07853890701232057

[47] Rosenberg J, Gustafsson F, Remme WJ, Riegger GA, Hildebrandt PR (2008) Effect of beta-blockade and ACE inhibition on B-type natriuretic peptides in stable patients with systolic heart failure. Cardiovasc Drugs Ther 22: 305–311. 18309461 10.1007/s10557-008-6099-6

[48] Ohtsuka T, Hamada M, Hiasa G, Sasaki O, Suzuki M, et al (2001) Effect of beta-blockers on circulating levels of inflammatory and anti-inflammatory cytokines in patients with dilated cardiomyopathy. J Am Coll Cardiol 37: 412–417. 11216955 10.1016/s0735-1097(00)01121-9

[49] Nishio M, Sakata Y, Mano T, Ohtani T, Takeda Y, et al (2008) Beneficial effects of bisoprolol on the survival of hypertensive diastolic heart failure model rats. Eur J Heart Fail 10: 446–453. 18400557 10.1016/j.ejheart.2008.03.002

[50] Bartholomeu JB, Vanzelli AS, Rolim NP, Ferreira JC, Bechara LR, et al (2008) Intracellular mechanisms of specific beta-adrenoceptor antagonists involved in improved cardiac function and survival in a genetic model of heart failure. J Mol Cell Cardiol 45: 240–249. 18632114 10.1016/j.yjmcc.2008.05.011

